# Colorless and transparent polyimide nanocomposites using organically modified montmorillonite and mica

**DOI:** 10.1038/s41598-024-61331-9

**Published:** 2024-05-09

**Authors:** Sanghyeon Park, Changyub Na, Sung-Soo Kang, Lee Ku Kwac, Hong Gun Kim, Jin-Hae Chang

**Affiliations:** 1 Graduate School of Carbon Convergence Engineering, Jeonju University, Jeonju, 55069 South Korea; 2 Institute of Carbon Technology, Jeonju University, Jeonju, 55069 South Korea

**Keywords:** Materials science, Nanoscience and technology

## Abstract

In this study, we introduce a method for replacing the glass used in existing display electronic materials, lighting, and solar cells by synthesizing a colorless and transparent polyimide (CPI) film with excellent mechanical properties and thermal stability using a combination of new monomers. Poly(amic acid) (PAA) was synthesized using dianhydride 4,4′-biphthalic anhydride (BPA) and diamine 2,2-bis(3-amino-4-hydroxyphenyl) hexafluoropropane (AHP). Various contents of organically modified montmorillonite (MMT) and mica were dispersed in PAA solution through solution intercalation, and then CPI hybrid films were prepared through multi-step thermal imidization. The organoclays synthesized to prepare CPI hybrid films were Cloisite 93A (CS-MMT) and hexadimethrine-mica (HM-Mica) based on MMT and mica, respectively. In particular, the diamine monomer AHP containing a –OH group was selected to increase the dispersibility and compatibility between the hydrophilic clays and the CPI matrix. To demonstrate the characteristics of CPI, the overall polymer structure was bent and a strong electron withdrawing –CF_3_ group was used as a substituent. The thermomechanical properties, morphology of clay dispersion, and optical transparency of the CPI hybrid films were investigated and compared according to the type and content of organoclays. Two types of organoclays, CS-MMT and HM-Mica, were dispersed in a CPI matrix at 1 to 7 wt%, respectively. In electron microscopy, most of the clays were uniformly dispersed in a plate-like shape of less than 20 nm at a certain critical content of the two types of organoclays, but agglomeration of the clays was observed when the content was higher than the critical content. Hybrids using HM-Mica had better thermomechanical properties and hybrids containing CS-MMT had better optical transparency.

## Introduction

As a condition of a substrate suitable for a flexible display, there should be no change in device characteristics even if the shape of the substrate is changed or bent, and it should have characteristics that are resistant to humidity and do not dissolve in solvents^1,2^. However, glass substrates, which have been widely used in conventional displays, have the disadvantages of being heavy, brittle, inflexible, and difficult to roll-to-roll process. Therefore, research is continuously being conducted on polymer materials suitable for electronic materials, including flexible displays that can replace glass^3,4^.

Recently, many studies have been published on the application of aromatic polyimides (PIs) having light weight, chemical resistance, and dimensional stability to electronic materials such. Although many types of aromatic PIs have excellent physical properties, it is difficult to apply them to display fields that require colorless and transparent optical properties due to their deep yellow color^5–7^. Colorless and transparent PI (CPI) can be applied to liquid crystal displays (LCDs) due to its low refractive index and birefringence. It is colorless and transparent with excellent mechanical properties and thermal stability, making it suitable for use as a semiconductor insulating film, thin film transistor (TFT-LCD) insulating film, passivation film, liquid crystal alignment film, material for optical communication, and protective film for solar cells. It can be utilized in various fields such as anti-reflection films and flexible display substrates.

The dark brown colors commonly seen in PI films are due to the charge transfer complex (CTC) phenomenon^8,9^. In order to block CTC appearing in aromatic PI and synthesize new CPI, the following various methods are mainly used: (1) In order to limit the movement of π electrons in the chain, functional groups such as trifluoromethyl (–CF_3_), sulfone (–SO_2_), and ether (–O–) with strong electronegativity are introduced into the main chain. (2) In order to prevent the movement of π electrons present in the main chain, a *meta*-substituted monomer structure is introduced into the main chain to bend the entire polymer structure^10–12^. (3) Recently, an attempt to introduce an alicyclic structure has been announced. Using these alicyclic monomers can greatly reduce the CTC effect and improve the film transmittance due to the alicyclo-ring structure^13,14^. In order to synthesize CPI, since the main chain must have a bent structure or an asymmetric substituent, although the colorless and transparent properties are satisfactory, the physical properties of CPI rapidly decrease. In order not to degrade the overall physical properties of CPI while maintaining its optical transparency, it is necessary to design a new monomer structure and introduce an innovative hybrid material concept^15,16^.

The polymeric nanocomposite refers to a hybrid in which fillers are uniformly dispersed in a nano-size within a polymer matrix^17,18^. Recently, fillers frequently used in nanocomposites include clay, carbon nanotubes, and graphene, etc.^19–21^. Among them, when clay is used as a filler, it is possible to manufacture a hybrid material in which polymer chains between clay layers can be completely exfoliated in nano-size. Clay has unique properties such as thermal stability, stiffness, and barrier properties due to its unique form and dispersibility, so dispersing an appropriate amount of clay in pure polymer can greatly improve physical properties due to orientation of rigid plate-like clay sheets^22,23^. Commonly used smectite-based clays are classified into montmorillonite (MMT), saponite, hectorite, water-soluble bentonite, and mica, etc^24^. For a long time, several researchers presented a basic model in the relationship between the structure and dispersion of various pristine clays and explained the effect of mutual attraction on dispersion. Clay with a surface area of approximately 700–800 m^2^/g greatly increases the thermomechanical properties of nanocomposites by using the mutual attraction between clay and polymer, even if a small amount is used^25–27^. In addition, if clays with a large aspect ratio (length/width, L/W) are perfectly dispersed in the polymer matrix, the heat resistance, solvent resistance, and insulating effects of the hybrids are also increased^28^. Especially, MMT and mica have excellent compatibility with polymers and are known to impart excellent thermal stability, tensile properties, and gas barrier properties to hybrid materials while maintaining their original optical properties^23,25^.

In general, hydrophilic pristine clays have an L/W ratio of about 200–2000 and are not compatible with lipophilic polymers^29^. Therefore, many experimental attempts have been made to improve the compatibility and dispersibility between raw clay and polymer. After all, it is widely known that organoclay synthesized by substituting the surface of clay with an appropriate organic modifier is uniformly dispersed in the form of nano-sized small particles in a polymer matrix in a hybrid^30,31^. In the case of most hybrids, the physical properties were greatly improved even when a small amount of less than 10 wt% of clay was added to the polymer used as a matrix. In several studies already published^28,29^, in the case of hybrids using clay, hybrids with the best physical properties were obtained at a specific critical content of clay, and some physical properties rather decreased when the clay content was above the critical content^32^.

In this study, PAA was synthesized using dianhydride 4,4′-biphthalic anhydride (BPA) and diamine 2,2-bis(3-amino-4-hydroxyphenyl)hexafluoropropane (AHP). Afterwards, organoclay was dispersed in PAA, and a new CPI hybrid film was synthesized through several stages of heat treatment. In order to improve the dispersibility and compatibility of lipophilic polymer and hydrophilic pristine clay, first, AHP monomer containing –OH group was used, and second, CPI hybrids were synthesized using Cloisite 93A (CS-MMT) and hexadimethrine-mica (HM-Mica) as organically modification clays.

The purpose of this study was to prepare various CPI hybrid films in which two types of organoclays were dispersed at various contents ranging from 1 to 7 wt% to synthesize new CPI hybrid films. Additionally, the thermomechanical properties, morphology based on clay dispersion, and optical transparency of the synthesized CPI hybrids were measured according to the type and content of the organoclay. Their physical properties were compared with each other. Furthermore, the critical content of organoclay that represents the best physical property value in the hybrid was also investigated.

## Methods

### Materials

CS-MMT organically modified with MMT was purchased from Southern Clay product, Co. (Tokyo, Japan). CS-MMT was synthesized by chemically substituting an alkyl group with MMT^33,34^. HM (MW = 4,000–6,000) was purchased from Sigma Aldrich (Yongin, Korea). HM-Mica was synthesized directly in our laboratory through several steps using HM^35^. The cation exchange capacities (CEC) of MMT and mica were 119 and 80 meq/100 g, respectively^25^. Monomers BPA and AHP required for CPI synthesis were purchased from TCI (Tokyo, Japan). *N,N*′-dimethylacetamide (DMAc) used as a solvent for CPI synthesis was purchased from Sigma Aldrich (Yongin, Korea) and moisture was removed using a molecular sieve (4 Å).

### Preparation of the CPI hybrid film

**Figure 1:**
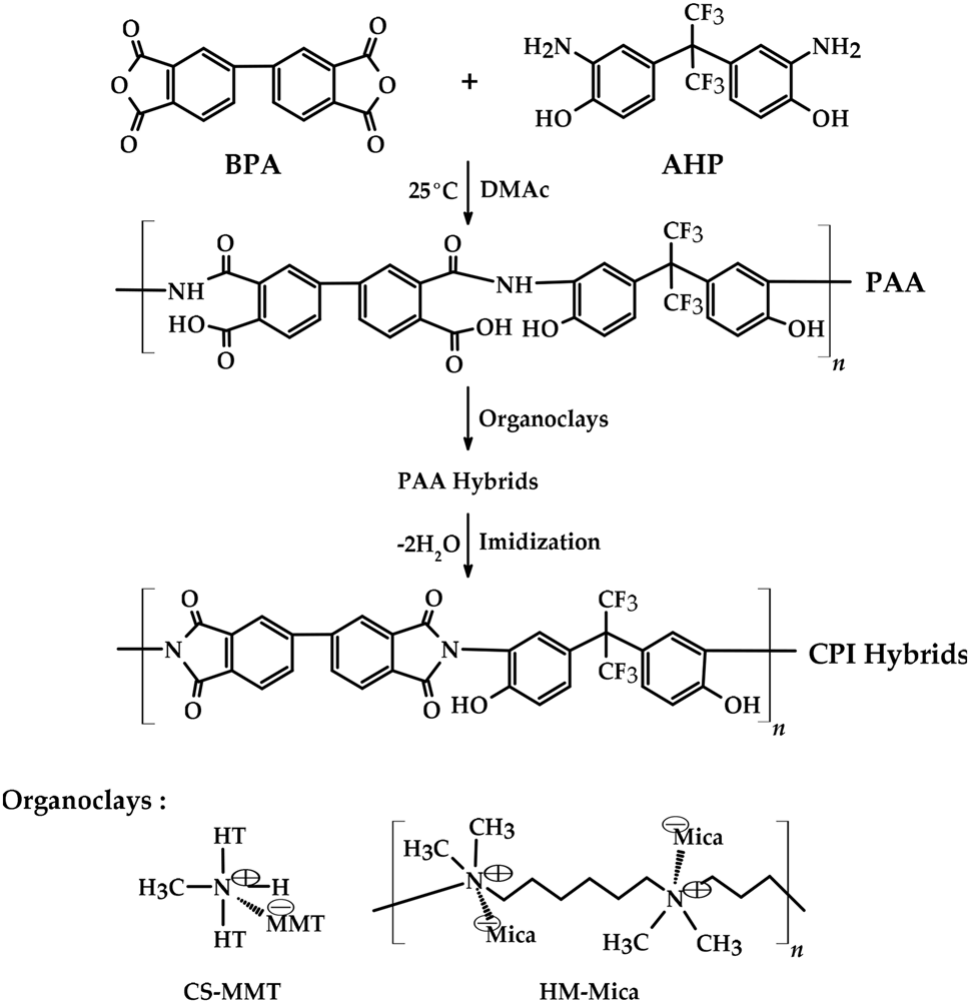
Synthesis route for the fabrication of CPI hybrid films.

PAA was synthesized from BPA and AHP using DMAc under low temperature conditions as follows: PAA was synthesized by dissolving BPA (2.94 g, 1.02 × 10^−2^ mol) and AHP (3.66 g, 1.02 × 10^−2^ mol) in 40 mL of DMAc and stirring under nitrogen conditions for 16 h. For the synthesis of the CPI hybrid film, the organoclays CS-MMT and HM-Mica were used in the range of 1 to 7 wt% compared to the matrix. Since the dispersion methods were all the same, only the case of 1 wt% of CS-MMT will be given as an example: 0.064 g of CS-MMT was dispersed in 30 mL of DMAc and stirred for 2 h. The dispersed CS-MMT solution was mixed with the PAA solution, allowed to rest for 30 min, and then sonicated for 1 h. This process was repeated three times. To remove DMAc in the PAA hybrid solution, it was vacuum dried using a glass plate for 2 h at 50 °C and 80 °C, respectively. Subsequently, the PAA hybrid was heat treated for various times starting at 110 °C and finally reaching 235 °C. The specific stepwise heat treatment conditions to obtain the CPI hybrid films are summarized in Table 1. After the reaction was completed, the CPI hybrid film was taken out of the vacuum oven, maintained at room temperature, and then removed from the glass plate in a 3% hydrofluoric acid (HF) aqueous solution. In order to increase the reliability of the measured value, the thickness of all films was kept constant in the range of 31–35 μm. The overall CPI hybrid synthesis route using organoclays is also shown in Fig. 1. Synthesis of a CPI hybrid film containing more than 8 wt% (0.512 g) organoclay was also attempted, but air bubbles occurred during polymerization and the film broke into several pieces. From this result, it is believed that excessive clay content hinders the formation of CPI hybrid films.

**Table 1:** Heat treatment conditions of the CPI hybrid film.

Sample	Temperature (℃)/time (h)/Pressure (torr)
PAA	25/16/760
PAA hybrid	50/2/1 → 80/2/1
CPI hybrid	110/1/1 → 140/1/1 → 170/1/1 → 195/0.8/1 → 220/0.8/1 → 235/2/1

### Characterization

For clay dispersion, an ultrasonic cleaner (WUC-D06H, Daehan Sci., Wonju, Korea) was used under the conditions of 425 watts and 40 kHz. Fourier transform-infrared (FT-IR; PerkinElmer, Spectrum Two, Llantrisant, UK) spectroscopy and solid-state ^13^C cross-polarized /magic angle spinning nuclear magnetic resonance (CP/MAS NMR; AVANCE II+, Bruker, Berlin, Germany) spectrometer were used to confirm the chemical structure of the synthesized CPI. The samples in cylindrical zirconia rotors were spun with a spinning rate of 12 kHz for the CP/MAS NMR experiments. Chemical shift was referenced to tetramethylsilane (TMS) for ^13^C as standard materials in order to obtain the accurate NMR chemical shift.

Wide-angle X-ray diffraction (XRD; Rigaku D/Max-IIIB, Tokyo, Japan) of CPI and hybrid films was measured using X-ray diffractometer equipped with Ni-filtered Cu-Kα target at room temperature. The morphology of the hybrid intercalated between clay layers was observed using a transmission electron microscope (TEM; JEOL, JEM 2100, Tokyo, Japan). The samples were cured in epoxy resin at 70 °C for 24 h and then sliced into 90 nm thickness with a microtome in vacuum. The accelerating voltage for TEM measurement was maintained at 120 kV.

A differential scanning calorimeter (DSC; 2-00915, Delaware, USA) and a thermogravimetric analyzer (TGA; SDT 0650-0439, Delaware, USA) were used at a heating rate of 20 °C/min under nitrogen conditions. To determine the coefficient of thermal expansion (CTE) of the CPI hybrids containing various organoclay contents, the thermal expansibility of the CPI hybrid film was observed using a thermomechanical analyzer (TMA, SS6100, Tokyo, Japan). The heating rate and expansion force at the time of measurement were maintained at 5 °C/min and 0.1 N, respectively, and the resulting values were obtained through secondary heating in the temperature range of 50–200 °C.

The mechanical tensile properties were tested using a universal tensile machine (UTM; Shimadzu, JP/AG-50KNX, Tokyo, Japan) with a crosshead speed of 5 mm/min. At least 10 measurements were conducted for each sample, and any values outside the error range were disregarded before averaging the remaining results.

To investigate the optical transparencies were measured using a UV–vis. spectrometer (Shimadzu UV-3600, Tokyo, Japan). The yellow index (YI) of the CPI hybrid film was measured with a spectrophotometer (Minolta CM-3500D, Tokyo, Japan). The observation angle was measured at 10° and a CIE-D light source was used.

### Experimental results

#### FT-IR spectroscopy

**Figure 2:**
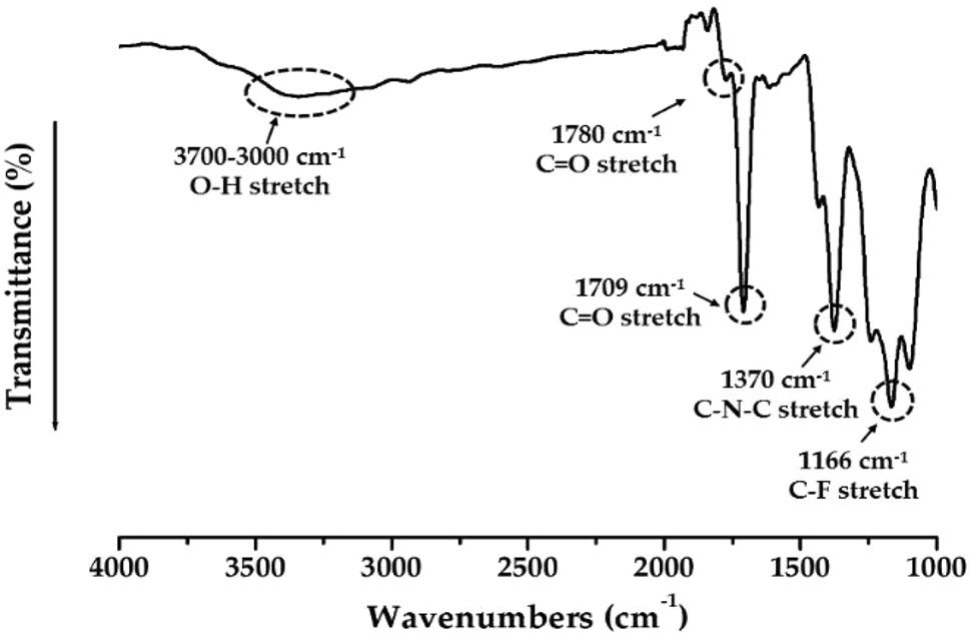
FT-IR spectrum of CPI.

Figure 2 shows the FT-IR spectrum of the synthesized CPI. The stretch peak, which can confirm the presence of –OH group, was widely observed at 3700–3000 cm^−1^. The C=O stretching peaks appeared at 1780 and 1709 cm^−1^, and the C–F stretching peak was observed at 1166 cm^−1^. Since the C–N–C peak, which is a CPI characteristic peak, was observed at 1370 cm^−1^, it was confirmed that CPI was perfectly synthesized from PAA by heat treatment^36^.

**Figure 3:**
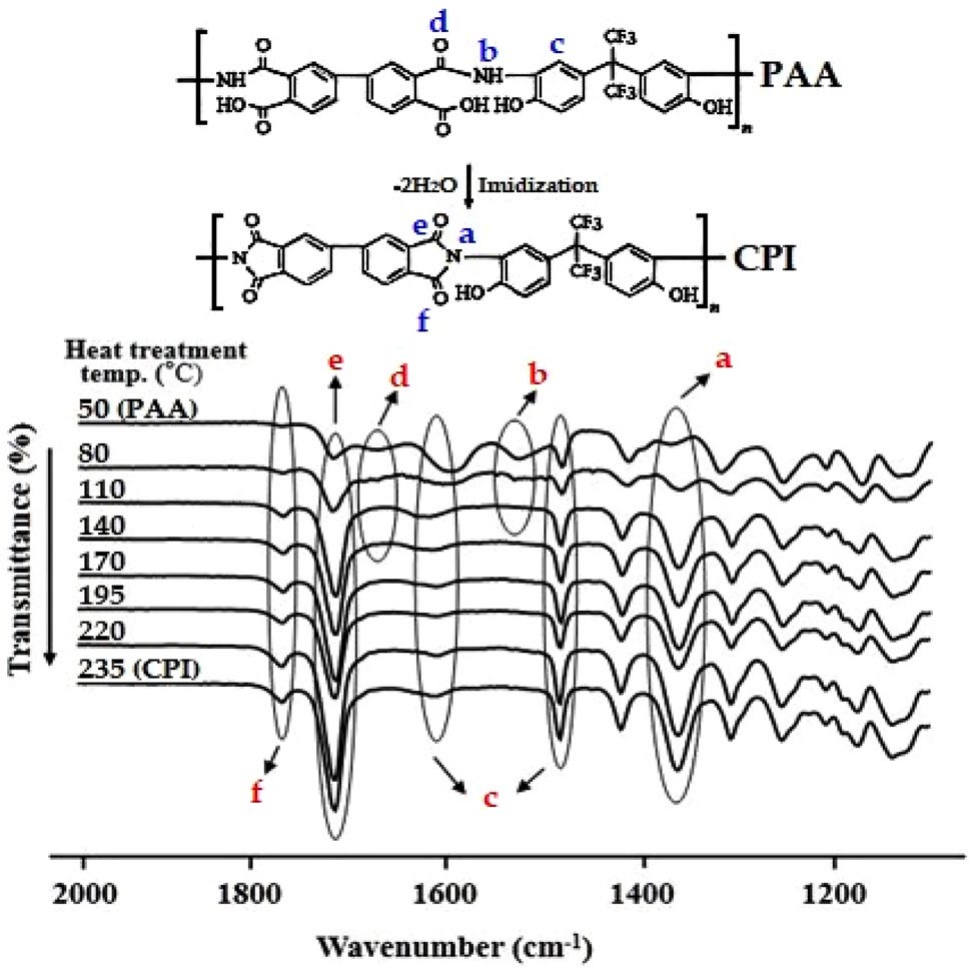
FT-IR spectra of CPI were recorded under different heat treatment temperatures.

To ascertain whether PAA transformed into PI under various heat treatment conditions, the CPI structure was analyzed based on the functional group peaks, as illustrated in Fig. 3. In PAA, at the 50 °C heat treatment condition, an amide C=O stretching peak at 1684 cm^−1^ (peak *d*) and an amide NH bending peak at 1536 cm^−1^ (peak *b*) were observed. However, these two peaks vanished as the heat treatment temperature increased. Moreover, the intensity of the C=O stretching peaks observed at 1780 cm^−1^ (*f*) and 1709 cm^−1^ (*e*), along with the C–N–C stretching peak observed at 1370 cm^−1^ (*a*), progressively increased as the annealing temperature rose to 235 °C. Thus, it can be affirmed that the imidization reaction of PAA took place at the various heat treatment temperatures.

#### NMR spectroscopy

**Figure 4:**
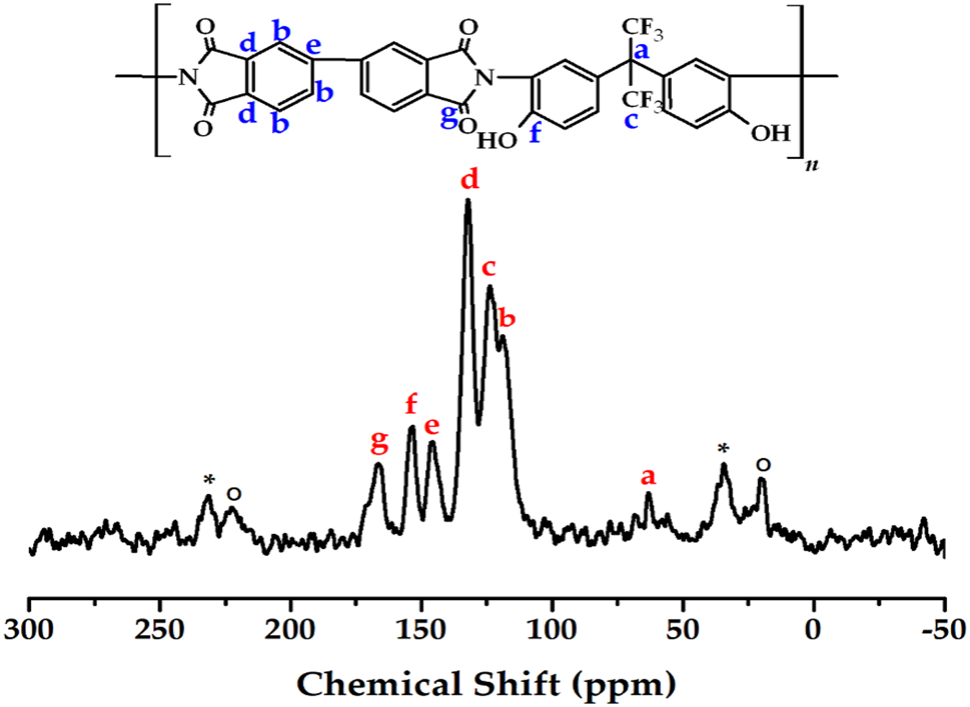
^13^C-NMR spectrum of CPI.

The chemical structure of CPI was confirmed using CP/MAS NMR, and the ^13^C NMR chemical shift corresponding to each peak is shown in Fig. 4. In the chemical shift of CPI, peaks corresponding to the phenyl ring were observed at 119.22 (*b*), 132.02 (*d*), and 145.98 (*e*) ppm. And ^13^C peaks of carbon connected to –CF_3_ and CF_3_ itself were observed at 63.28 (*a*) and 123.75 (*c*) ppm, respectively. Finally, the peak for ^13^C adjacent to –OH was recorded at 153.81 (*f*) ppm, and a peak corresponding to C=O of the imide functional group is observed at 166.61 (*g*) ppm^37^. Here, the sidebands for 132.02 ppm is remarked the asterisks (_*_) and those for 123.75 ppm is denoted the open circles (O). From these results, the chemical structure of the synthesized CPI was confirmed.

#### XRD pattern

**Figure 5:**
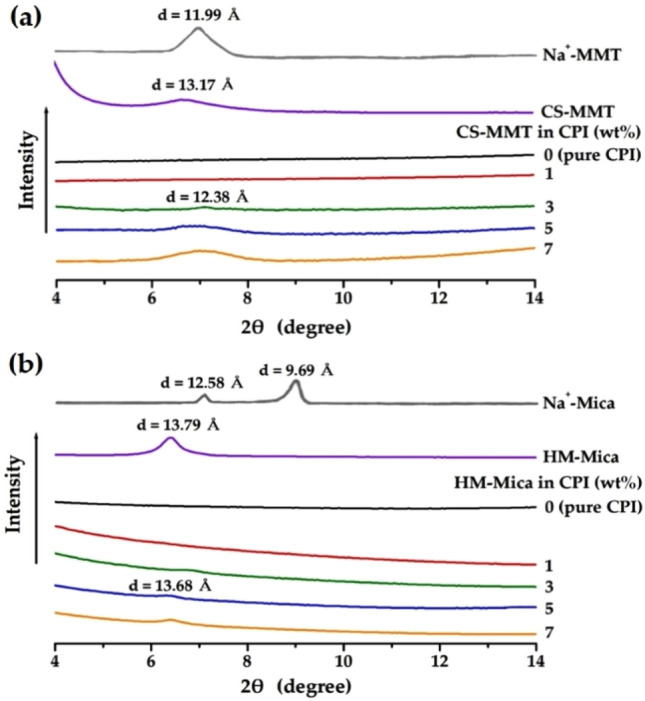
XRD patterns of CPI and CPI hybrid films with various organoclay contents. (**a**) CS-MMT and (**b**) HM-Mica.

Figure 5 shows the XRD patterns of pristine clays Na^+^-MMT, Na^+^-Mica, and organoclay and the XRD results obtained in the range of *2θ* values of 4–14° for the CPI hybrid film containing each organoclay content. The XRD peak of Na^+^-MMT shows a diffraction peak at *2θ* = 7.36° (*d* = 11.99 Å), whereas organoclay CS-MMT shows a peak at *2θ* = 6.65° (*d* = 13.17 Å) (see Fig. 5a). The increase in the interlayer spacing *d* in organoclay compared to pristine clay is due to the substitution of long alkyl chains on the clay surface. As the distance *d* between clay layers increases, intercalation of polymer chains between clay layers becomes easier, which can have a favorable effect on the formation of hybrids with excellent dispersibility^38^. Clay peaks in the CPI hybrid film were not observed until 1 wt% CS-MMT was dispersed. However, when 3 wt% (0.191 g) of CS-MMT was dispersed, a very small peak appeared at *2θ* = 7.11° (*d* = 12.38 Å), and this peak was observed at 7 wt% (0.446 g) with the same intensity at the same location. This phenomenon is because, as the clay content increases above the critical content, complete exfoliation inside the polymer matrix fails and new crystals are formed due to the agglomeration of excess clay^39,40^.

The characteristic peaks of pristine Na^+^-mica appeared as two strong peaks at *2θ* = 7.02° (*d* = 12.58 Å) and 9.12° (*d* = 9.69 Å), as shown in Fig. 5b. The longer *d* value of the interlayer spacing of pristine mica (*d* = 12.58 Å) than that of MMT (*d* = 11.99 Å) can be advantageous for dispersion because it is easier for the polymer chains to intercalate between the clay layers. The *d* value of organoclay HM-Mica (*d* = 13.79) was slightly increased compared to pristine mica. This result can be explained by the increase in the distance between clay layers due to the substituted organic material. No crystalline peaks were observed in the hybrid films when HM-Mica was dispersed up to 3 wt% in the same matrix. From this result, it was found that the clay was completely exfoliated from the matrix to form a nano-sized composite. However, at the 5 wt% (0.318 g) content, the clay aggregated and a very small peak appeared at *2θ* = 6.42° (*d* = 13.68 Å), and this peak was observed at the same position even when the clay content increased up to 7 wt%. As already described in CS-MMT, this can be seen as a result of the clay not being well dispersed in the CPI matrix and agglomerated with each other as the content of the organoclay increases. However, even when the two types of organoclays were used up to 7 wt%, the intensity of the XRD peak was very small, so it was expected that the clay aggregation would not be serious.

X-ray diffractometer is a good device for roughly observing the dispersion form of organoclay and measuring the *d* value, which is the distance between clay layers of dispersed hybrids. However, there are limitations in directly measuring the degree of dispersion of clay in a polymer matrix. In general, XRD is often used to check the simple agglomeration phenomenon of clay or the distance between layers, but TEM should be used to see more accurate morphology such as overall dispersion, agglomeration, and orientation of clay. In addition, it is necessary to observe the detailed morphology of the clay dispersed in the matrix polymer and to confirm the difference in how different types of organoclays are dispersed differently in CPI.

#### TEM micrograph

Using TEM, it is possible to quantitatively know the actual interlayer structure of the dispersed clay and how well it is dispersed in the nanoscale within the polymer matrix, and it is possible to accurately know the information of the nanocomposite by complementing the XRD results^41,42^.

**Figure 6:**
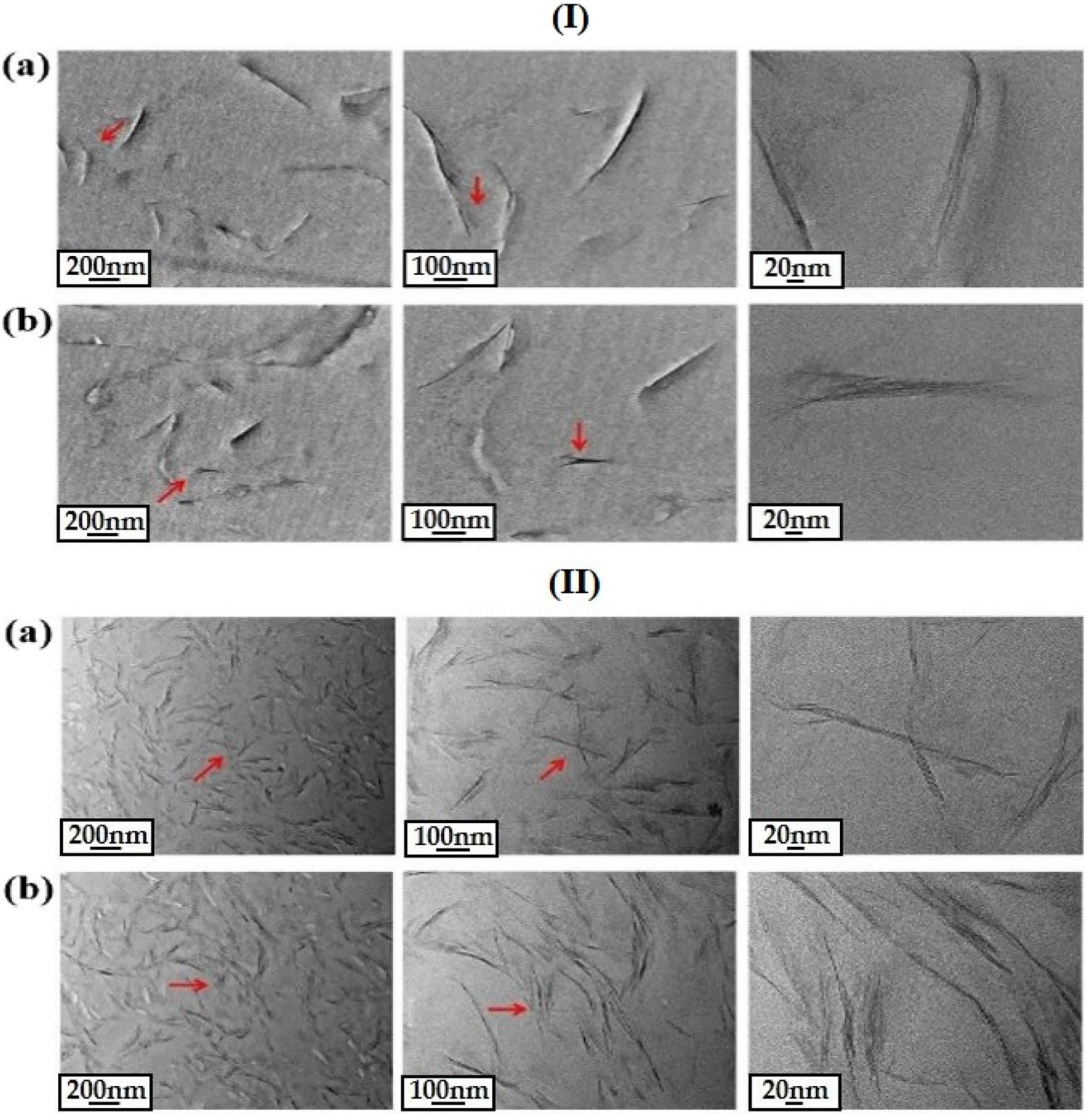
TEM photographs of CPI hybrid films containing (**a**) 3 and (**b**) 5 wt% for CS-MMT (I) and (**a**) 5 and (**b**) 7 wt% for HM-Mica (II) at various magnifications.

TEM was used to observe the dispersion of clay in the CPI matrix of hybrid films containing various contents of CS-MMT and HM-Mica, and the results are shown in Fig. 6. In each picture, hair-like black lines representing the 1-nm-thick clay layers could be seen, and the spaces between them represent the interlayer spacing between the clays. In addition, more detailed dispersion was observed by enlarging the area indicated by the arrow according to the magnification.

In the case of CS-MMT, when the content of organoclay was 3 wt% (Fig. 6I,a), it was confirmed that it was very well dispersed with a thickness of about 20 nm, and when enlarged, hair-like thread-like clay was clearly observed. However, when the content was increased to 5 wt% (Fig. 6I,b), the clay was observed to be more aggregated than 20 nm. These results have already been confirmed by the XRD results in Fig. 5. Although the organoclay was increased from 3 to 5 wt% and the clay was agglomerated, it was confirmed that nano-sized hybrids in which the clay was well dispersed with an average thickness of less than about 30 nm were formed in the CPI matrix as a whole.

Figure 6II shows the morphology of the dispersed clay according to the content of HM-Mica. Through the results of (Fig. 6II,a), the size of the averagely agglomerated clay particles at the 5 wt% content was found to be in a very well dispersed state with an average thickness of less than about 20 nm. Even in the case of 7 wt% HM-Mica, the clay was evenly dispersed throughout the matrix, and the clay was agglomerated to about ≥ 30 nm (see Fig. 6II,b). In the hybrid film containing two types of organoclays, agglomeration was observed above a certain critical content of clay, which was consistent with the XRD results of Fig. 5. However, the agglomeration phenomenon of the two types of clay was not serious above the critical content.

Overall, the TEM results of the two types of organoclays showed that the clays were well dispersed at the nano-scale in both hybrid films. In addition, it was confirmed that HM-Mica was better dispersed in the CPI matrix than CS-MMT at the same content. These results indicate that when the long alkyl group of hexadimethrine used in mica widens the gap (*d*) between the clays more than the alkyl group used in CS-MMT, the insertion of polymer chains dispersed in the clay can be readily increased, thereby effectively enhancing dispersion. This will act as a positive factor for the thermomechanical characteristics that will be discussed shortly^43^.

#### Thermal property

**Figure 7:**
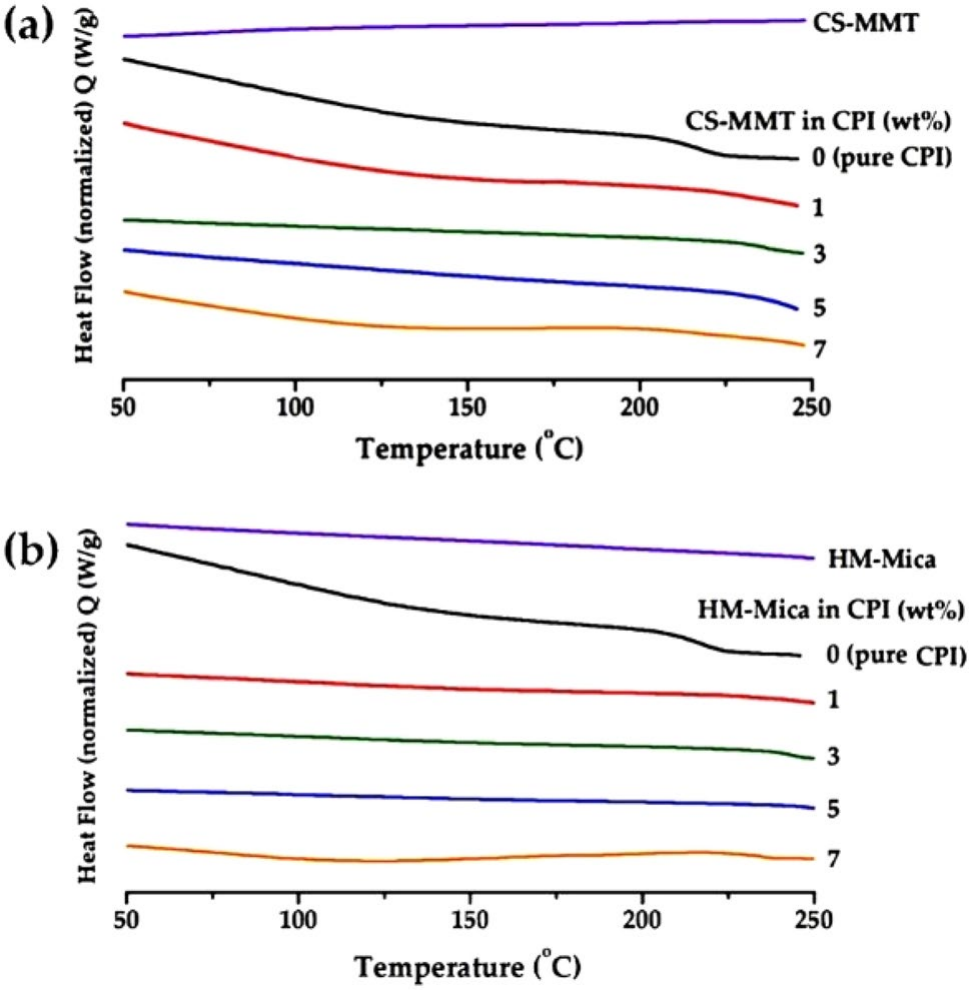
DSC thermograms of CPI and CPI hybrid films with various organoclay contents. (**a**) CS-MMT and (**b**) HM-Mica.

Table 2 summarizes the results of DSC of the CPI hybrid film using two types of organoclays, and the thermal behavior thereof is shown in Fig. 7. The glass transition temperature (*T*_*g*_) of pure CPI was 207 °C, but when the content of CS-MMT in the matrix was increased to 3 wt%, the *T*_*g*_ value increased to 229 °C. Generally, the increase in *T*_*g*_ is explained by two factors^44,45^. First, the rigid and plate-like clay layer dispersed in the polymer matrix significantly reduces the free volume of the intercalated polymer chains, so the *T*_*g*_ of the hybrid increase. The second factor is that the segmental movement of the polymer chains inserted inside the filler is hindered by the clay layer. In addition, *T*_*g*_ varies greatly depending on structural differences of monomers, secondary bonds such as hydrogen bonds included in polymer chains, fluidity of chains. Especially in the case of hybrids, it is directly affected by the type and content of filler^46,47^. And when the content of organoclay was increased to 7 wt%, the *T*_*g*_ rather decreased to 201 °C due to the aggregation of clay appearing above the critical content^48,49^. This result can already be explained by XRD and TEM results. (Figs. 5, 6). Similar to the case of CS-MMT, the *T*_*g*_ of the HM-Mica hybrid increased when the organoclay content was low, but decreased when the organoclay content was above the critical concentration. For example, when the HM-Mica content was increased to 5 wt%, the *T*_*g*_ increased to 243 °C. However, when the content of the organoclay was increased to 7 wt%, the *T*_*g*_ suddenly decreased to 216 °C. As already mentioned, the *T*_*g*_ decreased due to agglomeration of some clays above the critical content.

**Table 2:** Thermal properties of CPI hybrid films.

Organoclay in CPI (wt%)	CS-MMT				HM-Mica			
	*T*_*g*_^a^ (^°^C)	*T*_*D*_^*i*b^ (^°^C)	*wt*_*R*_^*600*c^ (%)	CTE^d^ (ppm/^°^C)	*T*_*g*_ (^°^C)	*T*_*D*_^*i*^ (^°^C)	*wt*_*R*_^*600*^ (%)	CTE (ppm/^°^C)
0 (pure CPI)	207	250	61	44.5	207	250	61	44.5
1	219	272	60	39.4	236	275	61	36.6
3	229	274	61	37.3	239	276	60	35.0
5	224	264	62	38.6	243	279	61	30.8
7	201	253	60	39.4	216	273	61	32.6

^a^Glass transition temperature.

^b^Initial decomposition temperature at 2% weight loss.

^c^Weight residue at 600 °C.

^d^Coefficient of thermal expansion for 2nd heating is 50 – 200 °C.

**Figure 8:**
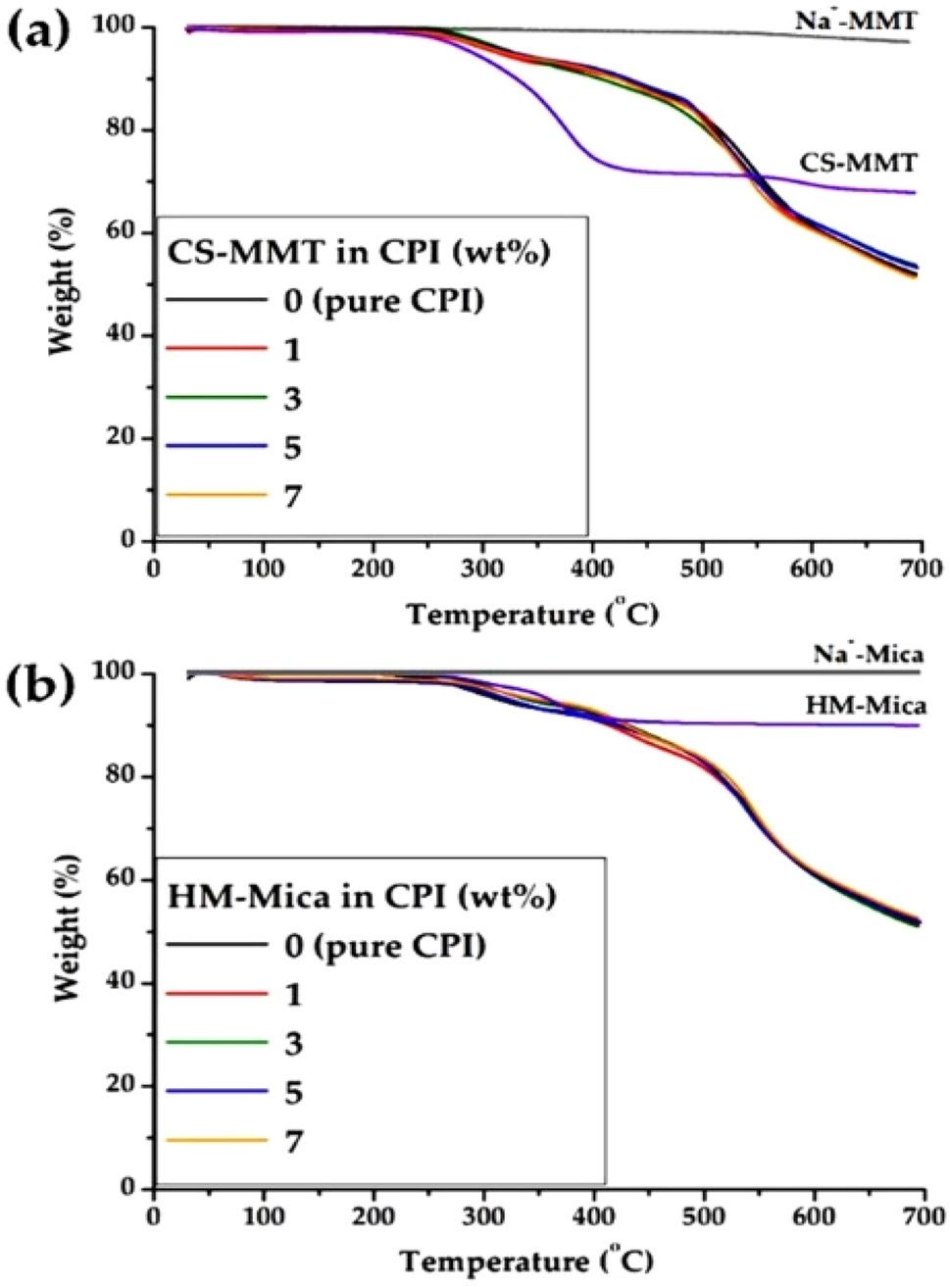
TGA thermograms of CPI and CPI hybrid films with various organoclay contents. (**a**) CS-MMT and (**b**) HM-Mica.

The behavior of the initial decomposition temperature (*T*_*D*_^*i*^), like that of *T*_*g*_, depended on various organoclay contents. Figure 8 shows the TGA thermograms of the CPI hybrid film containing various contents of the two types of organoclays, and their results are also summarized in Table 2. Two pure clays, such as MMT and Mica, showed excellent thermal stability that did not decompose even when heated up to 700 °C. However, organoclays showed an initial weight loss between about 250 and 400 °C (see Fig. 8).

**Figure 9:**
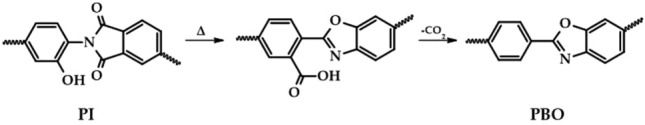
Mechanism by which PBO is formed from PI through thermal rearrangement.

These results can be explained by the low thermal stability of the chemically substituted alkyl groups in the clay. Also coincidentally, the *T*_*D*_^*i*^ of pure CPI started to decompose by 2% around 250 °C. The reason why the *T*_*D*_^*i*^ is lower than other PIs is because of the low thermal stability –OH group included in the main chain. As shown in Fig. 8, two thermal decomposition steps were observed upon heating in CPI and CPI hybrid synthesized using AHP monomer containing –OH group. This is because CPI with –OH group undergoes thermal rearrangement (TR) during heating to change to polybenzoxazole (PBO). Figure 9 shows the mechanism of converting CPI containing –OH group into PBO through TR^50,51^. Therefore, the decomposition of PBO started around 500 °C.

A large change in the *T*_*D*_^*i*^ value of the hybrid was observed even when a small amount of both types of organoclays was used. For example, when the content of CS-MMT was dispersed from 0 to 3 wt%, the *T*_*D*_^*i*^ increased from 250 to 274 °C, and when the content of HM-Mica was increased to 5wt%, the *T*_*D*_^*i*^ of the hybrid showed the highest value of 279 °C. These results are explained by the fact that the *T*_*D*_^*i*^ increased because the rigid plate-shaped clay effectively blocked the heat transfer path as it was evenly dispersed in the CPI matrix and suppressed volatilization of the CPI component when a high temperature was applied^52,53^. And when the content of each organoclay was increased to 7wt%, both *T*_*D*_^*i*^ values decreased regardless of the type of organoclay. This result seems to show that the effect of thermal stability is not properly exerted because the clay is not evenly dispersed in the CPI matrix and agglomerates with each other as the content of organoclay goes higher than the critical content. This result is also consistent with the previously described *T*_*g*_ trend.

The residual weight at 600 °C (wt_R_^600^) of pure CPI films and hybrids showed mostly constant values of 60 to 62%, regardless of the type and content of clay as shown in Table 2. This is because the low heat stability organic-alkyl groups in the organoclay are all decomposed at high temperatures and the remaining plate-shaped inorganic silicate clay layer itself has high heat resistance.

When a pure polymer is heated, it relaxes mainly in a direction perpendicular to the main chain. However, in the case of a hybrid in which hard and rigid plate-shaped clay is evenly dispersed, deformation by heating becomes difficult^54^. Therefore, the clays dispersed in the matrix polymer can suppress the lateral thermal expansion of the polymer due to heat because they have high thermal stability and can efficiently block heat transfer. Therefore, in order for the hybrid to have low thermal expansion characteristics, the thermally stable clay must be evenly dispersed in the polymer matrix. In polymers used as electronic materials, the thermal stability already mentioned is very important, and information on thermal expansion with temperature change also accounts for a large part of thermal stability^55,56^.

**Figure 10:**
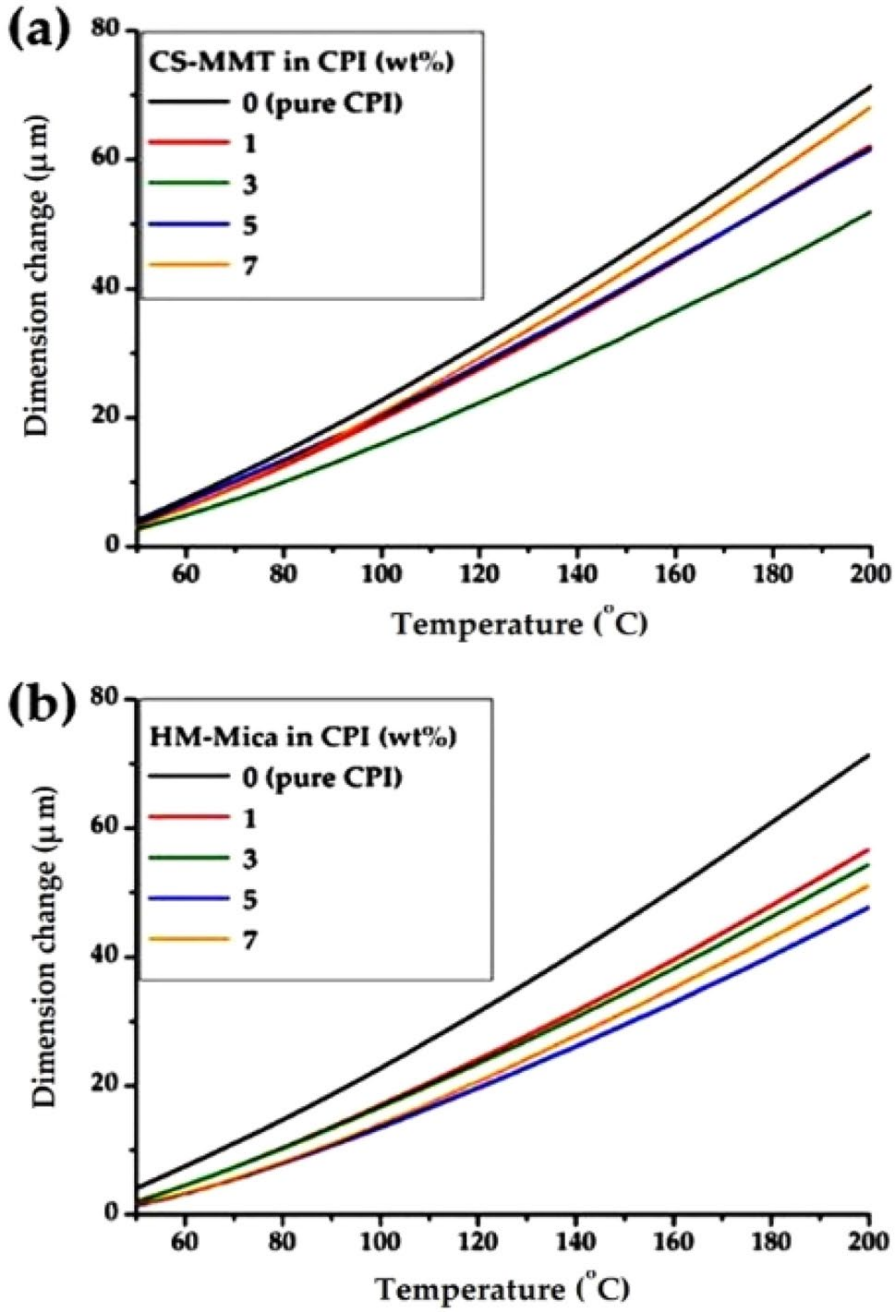
TMA thermograms of CPI and CPI hybrid films with various organoclay contents. (**a**) CS-MMT and (**b**) HM-Mica.

The thermomechanical analysis thermogram using TMA is shown in Fig. 10, and their summarized results are shown in Table 2. The thermal expansion coefficient is obtained using the formula below (ASTM E831):

$$ \Delta {\text{L}}\, = \,\alpha \cdot{\text{L}}\cdot\Delta {\text{T}}. $$

Here, ΔL is the change in length L, ΔT is the change in temperature, and α is the coefficient of linear expansion, which varies slightly with temperature^57^.

The CTE values were obtained by secondary heating of all CPI hybrid films in the temperature range of 50 to 200 °C to obtain reliable results. In the CPI hybrid, a change in CTE was observed depending on the filler content. That is, the CTE decreased from 44.5 to 37.3 ppm/°C from 0 to 3 wt% of CS-MMT, however, the CTE value also increased again to 39.4 ppm/°C as the filler content increased to 7 wt%. Unlike the case of CS-MMT, the change in CTE according to the filler content was greater in the HM-Mica hybrid than in the CS-MMT hybrid. The CTE values of the HM-Mica hybrid films decreased until a critical content was reached and then increased at higher filler content. For example, the CTE value decreased from 44.5 to 30.8 ppm/℃ as the HM-Mica content increased from 0 to 5 wt%, but increased to 32.6 ppm/℃ as the HM-Mica content increased to 7 wt%. The trend of these CTE values was consistent with the behavior obtained for *T*_*g*_ and *T*_*D*_^*i*^ values.

From the overall results, it was confirmed that HM-Mica was more effective than CS-MMT at the same content among the two types of organoclays used to increase the thermal properties of CPI. In particular, the hybrid film using CS-MMT showed a critical content at 3 wt%, but HM-Mica showed the highest thermal properties at 5 wt%. These results can be explained by the difference in L/W values between Mica and MMT. That is, as already explained in the introduction, the aspect ratios of mica and MMT are 1230 and 213, respectively, because the wide plate-shaped mica reduces the thermal effect from the outside and shows excellent heat resistance. In addition, as can already be seen in the XRD results (Fig. 5), because the interlayer distance *d* of mica is larger than that of MMT, the dispersibility of the polymer chains inserted between mica is better. This improved dispersion increases the thermal properties of the polymer chains uniformly intercalated within the clay layer.

#### Mechanical tensile property

Recently, a lot of research has been focusing on materials for flexible electronic devices, flexible semiconductors, and flexible displays. The outcomes of these studies show that materials with excellent optical transparency and thermomechanical properties can be applied to electronic components that can be bent or folded freely. Additionally, there will be an increasing demand for efficient and cost-effective roll-to-roll processes.

The mechanical properties of CPI hybrid films obtained with various organoclay contents were determined in terms of ultimate strength, initial modulus, and elongation at break. As already observed in the thermal properties, the mechanical properties also showed a maximum value at a certain critical content of filler, but decreased above the critical content. The detailed mechanical properties of hybrid films with various organoclay contents are summarized in Table 3.

**Table 3:** Mechanical properties of CPI hybrid films.

Organoclay in CPI (wt%)	CS-MMT			HM-Mica		
	Ult. Str.^a^ (MPa)	Ini. Mod.^b^ (GPa)	EB^c^ (%)	Ult. Str (MPa)	Ini. Mod. (GPa)	EB (%)
0 (pure CPI)	29	1.98	2	29	1.98	2
1	42	2.55	2	42	3.54	2
3	44	3.70	1	47	3.54	1
5	24	3.43	1	60	3.94	2
7	14	1.53	1	35	2.56	1

^a^Ultimate strength.

^b^Initial modulus.

^c^Elongation at break.

**Figure 11:**
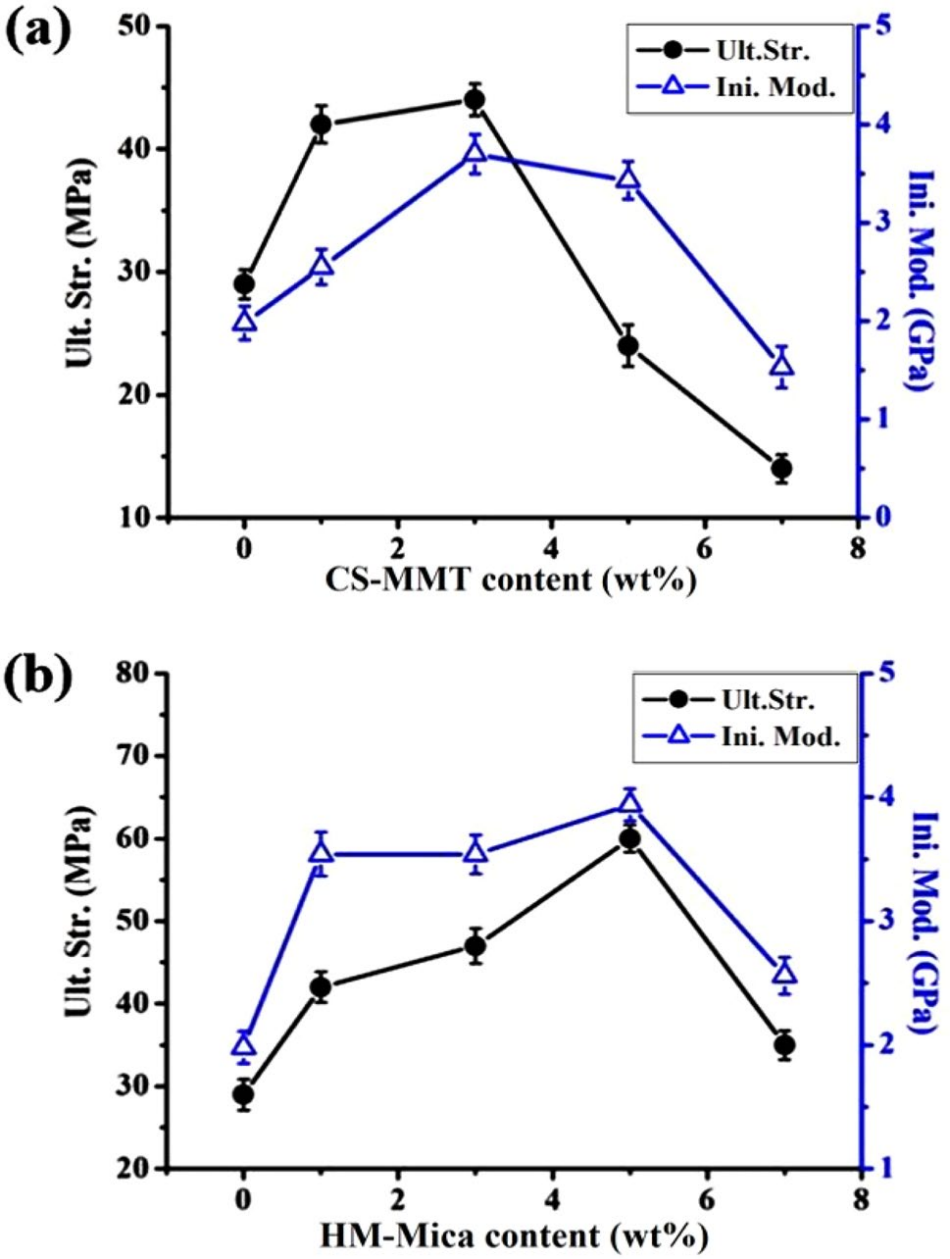
Mechanical tensile properties CPI hybrid films with various organoclay contents. (**a**) CS-MMT and (**b**) HM-Mica.

When the organoclay CS-MMT increased from 0 to 3 wt%, the ultimate strength and initial modulus increased from 29 to 44 MPa and from 1.98 to 3.70 GPa, respectively. In the case of HM-Mica hybrid, the mechanical properties showed the highest value when the organoclay content was 5 wt%. In other words, compared to pure CPI, the ultimate strength increased by about 107% (60 MPa) and the initial modulus increased by about 99% (3.94 GPa). The dispersion and orientation of fillers within the polymer matrix have a critical impact on the mechanical properties of hybrid films. This improvement in mechanical properties is due to the strength of the uniformly dispersed clay itself and the affinity of the clay with the matrix^58^. However, when the content of the two organoclays in the matrix reached 7 wt%, both the final strength and initial modulus suddenly decreased. For example, in the case of CS-MMT hybrid, the tensile strength and initial modulus decreased to 14 MPa and 1.53 GPa, respectively, and similar results were shown in the case of HM-Mica hybrid film (35 MPa and 2.56 GPa). This is thought to occur because excess clay used beyond the critical content tends to agglomerate, resulting in lumps larger than nano size within the hybrid film^59,60^, and these phenomena have already been confirmed by XRD and TEM (Figs. 5, 6). The change in mechanical properties according to the content of the two organoclays is shown in Fig. 11. Unlike the previously obtained strength and modulus, the elongation at break (EB) of all hybrid films was 1–2% regardless of the type and content of organoclay due to the reinforcing effect of the rigid plate-like clay. For these reasons, it is attributed to the synthesized CPI being not flexible or to the inherently brittle nature of the clay used in the hybrid film.

When comparing the effects of the two clays on overall mechanical tensile properties, organo-Mica showed significantly better results than organo-MMT. The reason for this, as already described in detail in the thermal properties, is that the dispersion and orientation of the clay are excellent due to the L/W and interlayer distance *d* values of Mica that are larger than those of MMT, allowing it to better withstand external tensile force.

The increase in thermomechanical properties of the CPI hybrid is closely related to the excellent dispersibility and orientation of the nanofillers added to the matrix, as well as the interlayer distance of the clay, and the interaction at the interface between the clay and the polymer. This affinity that may arise between the polymers and clays that make up the hybrid has a decisive impact on functions such as the transformation of the stress applied to the nanoparticles dispersed in the polymer matrix, changes in energy dissipation and energy storage in the matrix, and the viscoelastic response of the hybrid structure.

#### Optical transparency

As the amount of plate-shaped clay dispersed in the polymer matrix increased, the light transmittance of the hybrid film decreased, suggesting that light transmittance depends on the clay content. The optical transparency of the CPI hybrid films was evaluated using the cut-off wavelength (λ_o_), which refers to the initially transmitted wavelength, the transmittance at 500 nm (500 nm^trans^) in the visible light region, and the YI.

**Figure 12:**
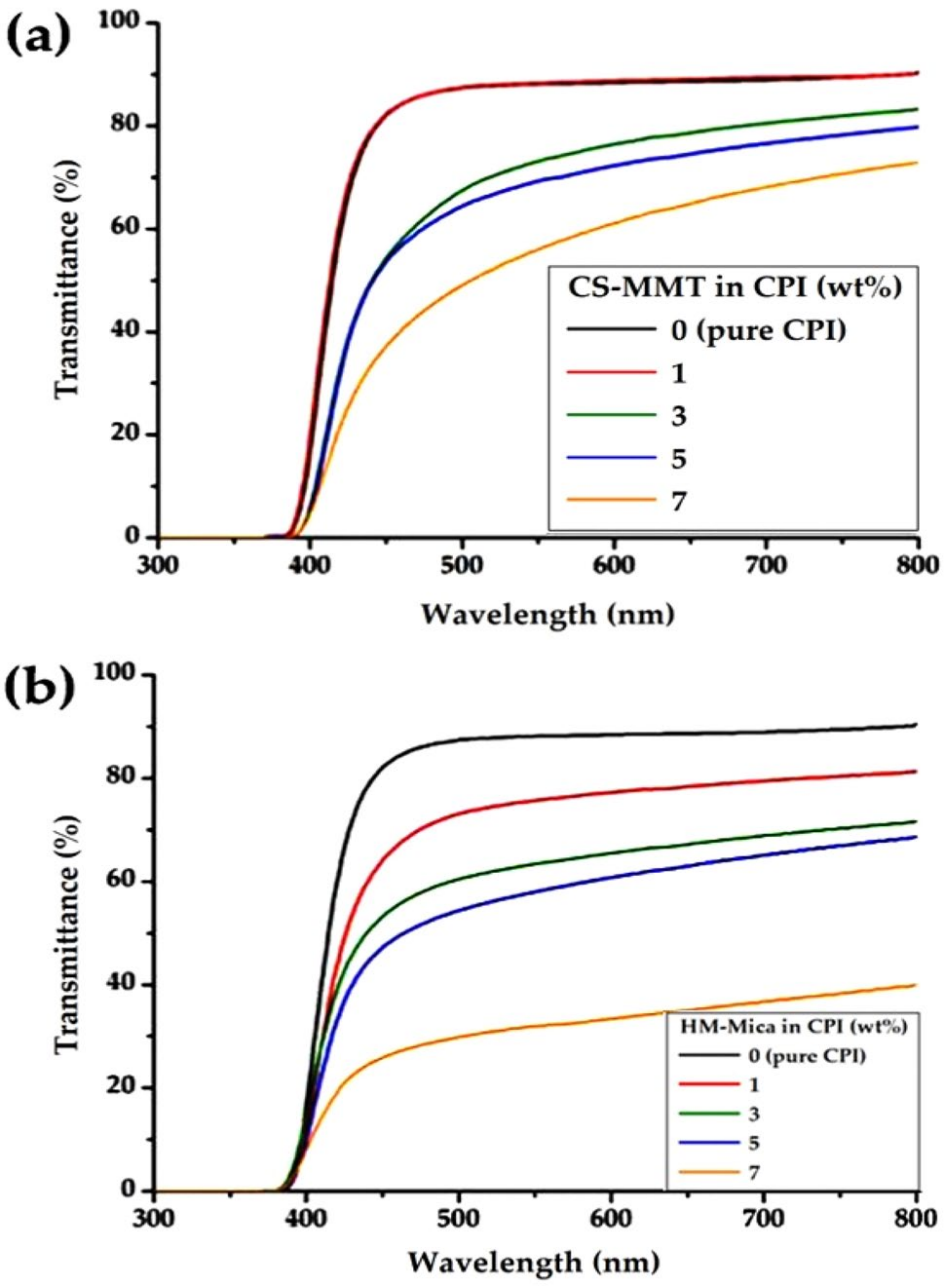
UV–Vis. transmittances of CPI and CPI hybrid films with various organoclay contents. (**a**) CS-MMT and (**b**) HM-Mica.

The UV-vis. spectra of the hybrid films are shown in Fig. 12, and the results are summarized in Table 4. The thickness of each hybrid film was maintained constant at 31–35 μm to compare their optical properties under the same conditions. The λ_o_ of the CPI hybrid was almost constant in the range of 388–392 nm regardless of the type and content of organoclay. This means that light is transmitted before reaching the visible ray region, and the optical transparency of the film is excellent.

**Table 4:** Optical properties of CPI hybrid films.

Organoclay in CPI (wt%)	CS-MMT				HM-Mica			
	Thickness^a^ (μm)	λ_0_^b^ (nm)	500 nm^trans^ (%)	YI^c^	Thickness (μm)	λ_0_ (nm)	500 nm^trans^ (%)	YI
0 (pure CPI)	33	388	87	5	33	388	87	5
1	35	390	87	8	31	388	73	9
3	34	392	68	11	31	389	60	13
5	35	392	62	13	34	390	54	15
7	35	392	49	15	35	388	30	18

^a^Film thickness.

^b^Cut-off wavelength.

^c^Yellow index.

However, the 500 nm^trans^ values of the CPI hybrid films decreased significantly with increasing clay content in both types of organoclays. That is, when the organoclay content increased from 0 to 7 wt%, the two types of hybrid films sharply decreased from 87 to 49% and 30%, respectively. In particular, HM-Mica had a larger decrease than CS-MMT. This decrease could be attributed to the plate-shaped clay dispersed in the CPI matrix, which hinders the transmission of light in the visible region. Additionally, since the aspect ratio value of mica is much larger than that of MMT, the decrease in 500 nm^trans^ is also presumed to be greatly affected by the plate aspect ratio.

**Figure 13:**
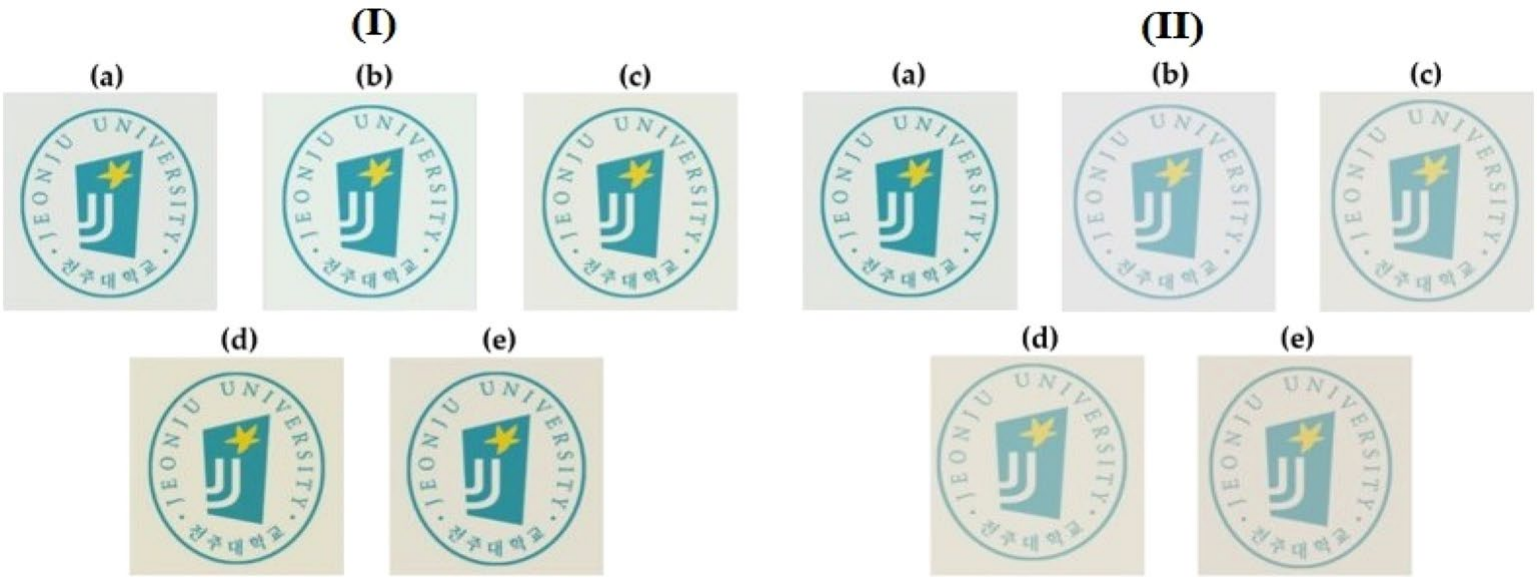
Photographs of CPI and CPI hybrid films with various organoclay contents. (**a**) 0 (pure CPI), (**b**) 1, (**c**) 3, (**d**) 5, and (**e**) 7 wt% for CS-MMT (I) and HM-Mica (II), respectively.

As shown in Table 4, the YI value was affected by the type and content of organoclay. As the content of the two types of organoclays increased, the YI of the hybrids also increased consistently. The YI of pure CPI was 5, but when CS-MMT and HM-Mica were used up to 7 wt% in the hybrid, the YI increased to 15 and 18, respectively. Overall, the YI value was higher when HM-Mica was used than CS-MMT in the hybrid film with the same content. This result can be explained by the aspect ratio of the clay already described above. Photographs of the pure CPI film and the CPI hybrid film with different types of organoclays are shown in Fig. 13. The hybrid films gradually turned darker as the organoclay content increased, but all films were clear and transparent, so there was no problem in reading the logo through the hybrid films.

## Conclusion

CPI hybrid film with excellent thermomechanical and optical properties is expected to be applied to electronic materials in the future. However, it is very challenging to maintain the optical transparencies of a polymer film while enhancing its thermomechanical properties by adding clay to the polymer matrix. In this study, these problems were systematically studied by dispersing organoclay as a filler in CPI hybrid films. In the future, applications such as flexible displays, flexible solar panels, and flexible printed circuit boards are expected to drive the market for excellent CPI films. This growth is attributed to the increased use of CPI films in the manufacturing of flexible displays. Over the past few years, significant advancements in the field of flexible active matrix organic light-emitting diode (AMOLED) displays, where hard glass is replaced by flexible substrates composed of CPI films, have made the potential of flexible displays a reality.

The thermal property, morphology, and optical transparency of CPI hybrid films obtained by dispersing 1 to 7 wt% of organoclay CS-MMT and HM-Mica in the CPI matrix were measured and their results were compared each other. The critical content of fillers that showed the highest values in the thermomechanical properties of the CPI hybrid films were 3 and 5 wt% for CS-MMT and HM-Mica, respectively. In addition, the thermomechanical properties of the hybrid film were superior when HM-Mica was used, but the optical transparency was better when CS-MMT was used. Based on this study, it is expected that by controlling the types and concentrations of nano-sized fillers dispersed in various structures of CPI matrices in the future, superior hybrid films with enhanced properties could be obtained.

As can be seen in our study, it is possible to synthesize high-performance CPI with excellent physicochemical properties that can be used under harsh conditions by accurately designing the monomer structure and appropriately controlling the reaction conditions. In addition, if nano-sized fillers are perfectly dispersed to obtain nanocomposite materials with better physical properties than conventional composite materials, many applications in the field of electronic- and display-materials will be possible in the future.

To lead the future, technologies such as nano thin film foundational technology, physical and chemical sensor material and device technology, and advanced materials science technology and device technology are required. To develop advanced materials using these technologies, sophisticated patterning and excellent physical and chemical properties must be the foundation. Therefore, the CPI hybrid obtained in the study could be a very appropriate alternative to improve the shortcomings of existing materials.

## Data Availability

The datasets used and/or analysed during the current study available from the corresponding author on reasonable request.
